# Rotation Rate Sensors and Their Applications

**DOI:** 10.3390/s21165344

**Published:** 2021-08-07

**Authors:** Zbigniew Zembaty, Felix Bernauer, Heiner Igel, Karl Ulrich Schreiber

**Affiliations:** 1 Faculty of Civil Engineering and Architecture, Opole University of Technology, ul. Prószkowska 76, 45-758 Opole, Poland; 2 Department of Earth and Environmental Sciences, Ludwig Maximilian University, Theresienstr. 41, 80333 Munich, Germany; fbernauer@geophysik.uni-muenchen.de (F.B.); igel@geophysik.uni-muenchen.de (H.I.); 3 Geodetic Observatory Wettzell, Research Unit Satellite Geodesy, Technical University of Munich, 93444 Bad Koetzting, Germany; ulrich.schreiber@tum.de

Measurements of rotations are unique because of their inherent property making them absolute and without an external frame of reference. This is in contrast to velocity, which needs an inertial system as a frame of reference. The Foucault pendulum has been a symbol of this fascination since it was publicly demonstrated in 1851. Despite all this, it took a long time before high-resolution rotation sensing became available in sensitive measurement devices. The necessity of navigation and attitude control for airplanes was the most important technology driver. After an era of mechanical gyroscopes in the first half of the last century, active optical Sagnac interferometers (ring lasers) became available in the 1970s, based on the advent of lasers in the early 1960s. These inertial measurement units were still fairly bulky, heavy, and delicate to handle. The need to reduce the size and weight led to the development of fiber optic gyroscopes (FOGs), which matured in the late 1980s. Although offering a moderate size and ease of operation, these devices were still very expensive due to the delicate manufacturing of the fiber coil and the photonic components inside. The next generation of rotation sensing devices exploits the Coriolis force on tiny solid-state proof masses etched from a silicon substrate. These micro-electro-mechanical systems (MEMS) can be mass-produced and come in a wide range of sensitivities. Tailored to specific needs, such MEMS gyroscopes are contained in every cellphone today.

Although there is an abundance of gyroscopes available on the market, very few of them are sensitive enough to quantitatively detect rotational signals (Love waves) from teleseismic activities, which requires a sensor resolution of less than 1 prad/s. Large ring lasers with enclosed sensor areas larger than 1 m^2^ are still the only instruments that offer this sensitivity [1]. However, in the regime of moderate earthquake motion, such as from smaller local earthquakes, FOGs with a sensitivity of about 10 nrad/s provide a convenient measurement range. From the measurements of seismic strong-motion to structural health monitoring, rotational sensors offer important properties because they can disentangle tilt from linear acceleration, and thus provide the true motion of a strap-down device fixed to a support structure, which may significantly deform under the excitation.

The situation for contemporary rotation sensing is very encouraging. There are a large number of sensor concepts available for applications, ranging from the detection of minute variations in the rotation rate of the Earth up to the strong-motion of several rad/s, and with high bandwidths. As diverse as the measurement applications are, the pool of available rotation sensing instruments is also varied.

Currently, rotation rate sensors are finding more and more applications in science and engineering, most prominently by serving as vehicle or airplane motion control. In consumer electronics, they are utilized for the tilt control of smartphones, whereas in modern seismology, their increased application has developed an emerging field of research: rotational seismology. With decreasing costs and increased sensitivity, rotation rate sensors can be effectively applied in structural health monitoring (SHM).

Accurate measurements of the spatial field of seismic ground motion are very difficult, and differentiation is particularly prone to noise pollution [2]; therefore, a need for the direct measurement of seismic rotations becomes obvious. Measuring rotational seismic ground motion became an emerging branch of seismology after the publication of the first Special Issue of the Bulletin of the Seismological Society of America [3], followed by the Special Issue of the Journal of Seismology [4]. During the past nine years, from 2012, this research field has grown remarkably. The idea of preparing a third Special Issue was proposed during the 5th workshop of the International Working Group on Rotational Seismology (https://www.rotational-seismology.org/), which took place at Sun Moon Lake, Taiwan, in September 2019, devoted to studying not only rotational seismology, but also relative fields of seismic engineering, geodesy, and structural health monitoring. Thus, the present Special Issue contains many contributions from the participants of that seminar.

Altogether, this Special Issue collects 19 papers on (inertial) rotation rate sensors and their applications. It focuses on the performance testing of novel rotation sensing techniques and pays particular attention to the novel areas of modern geophysics, including seismology and geodesy, seismic engineering, civil and mechanical engineering, including structural health monitoring (SHM). It is therefore fair to say that rotation rate sensors have substantially extended the inventory of 21st-century science and technology.

The first group of papers presented in this Special Issue are devoted to **constructing new measuring devices or their** **development**. These are the papers written by Cao et al. [5] or Muray-Bergquist et al. [6]. Modern geodesy and navigation are subjects of the papers by Rossi et al. [7] and Filatov et al. [8]. The paper by Brotzer et al. [9] describes enhancing the quality of data acquisition of one of the most sensitive rotation-rate measuring devices: a ring laser ROMY installed in Fürstenfeldbruck, Germany.

One prerequisite for accurate and reliable rotational motion observations is careful sensor performance characterization. For the majority of scientific domains discussed in this Special Issue, the application of rotation sensors is relatively new. Thus, at the time of writing, no standardized testing procedures exist for the instruments used. For that reason, this Special Issue dedicates ten contributions to the **testing and performance characterization of rotation sensors**. Bernauer et al. [10] describe a field experiment, which took place in Fürstenfeldbruck in November 2019, that was devoted to the comparison of 24 rotation rate sensors under artificially induced seismic excitations. The other three papers are devoted to more detailed field tests of particular devices during the Fürstenfeldbruck experiment: Kurzych et al. [11], Izgi et al. [12], and Brokešová et al. [13]. The paper by Cao et al. [5] focuses on the performance characteristics of specific seismic rotational motion sensors. The paper by Murray-Bergquist et al. [6] characterizes compact, strong-motion 6DoF sensor units for their future use in the seismic monitoring of buildings. Liu et al. [14] and Filatov et al. [8] discuss the performance of ring-laser gyroscopes as high-precision rotational motion measurement devices. Brotzer et al. [9] describe a novel and automated quality assessment procedure for ring-laser data and apply it to a data set recorded with the large ring-laser gyroscope ROMY located in Fürstenfeldbruck, Germany. Other field tests of rotational sensors were carried out in quarries and are described in the papers by Teisseyre et al. (2021) and Brokešová and Málek [15], where direct rotational motion measurements are compared with rotational motion observations derived from finite differencing across networks of classical seismic translation sensors. The paper by Fuławka et al. [16] describes an experiment measuring ground rotations using an R1 sensor installed in a deep copper-mining basin of LGOM in southwest Poland, excited by strong rock-bursts.

The third group of papers are devoted to the **application of rotational ground motion measurements in seismology**. The interest in observing rotational ground motions for seismic applications is not new. It was likely Schlüter who, at the beginning of the 20th century, was the first to suggest the possibility of estimating the seismic surface wave phase velocity from single-point measurements of vertical translational motion and transverse rotation (Beiträge zur Geophysik, vol. 5, 1903). However, these considerations remained mainly theoretical simply due to the lack of suitable instrumentation capable of measuring the low-amplitude rotational ground motions originating from seismic sources. Only recently (approximately, within the last decade) have instruments been developed that enable seismic rotational motion observations with very high precision. In addition, field-deployable seismic rotation sensors have opened a broad variety of applications to this new field of research. The problem of measuring surface rotations in seismology is worth a deeper introduction for the benefit of the reader of the Special Issue.

Consider an isolated point on the ground surface represented by an elastic half-space, which is the site of the measuring system. This instrument is capable of measuring motion in six degrees of freedom: three rotational (*ω_x_*(*t*), *ω_y_*(*t*), *ω_z_*(*t*)), in addition to three translational (*u_x_*(*t*), *u_y_*(*t*), *u_z_*(*t*)) degrees of freedom (Figure 1).

The classic elastic body dynamics equations relate the antisymmetric part of the spatial gradient tensor of the displacement field ***u***(*t*) to rigid body rotations **ω**(*t*), as follows:

(1) $$\left\{\begin{array}{c} \omega_{x}\\ \omega_{y}\\ \omega_{z} \end{array}\right\}=\frac{1}{2}\nabla \times u=\frac{1}{2}\left(\begin{array}{c} \partial_{y}u_{z}-\partial_{z}u_{y}\\ \partial_{z}u_{x}-\partial_{x}u_{z}\\ \partial_{x}u_{y}-\partial_{y}u_{x} \end{array}\right),$$

At the free surface, assuming stress-free boundary conditions and noting that our observables are rotational velocities, one obtains:

(2) $$\left\{\begin{array}{c} {\dot{\omega }}_{x}\left(t\right)\\ {\dot{\omega }}_{y}\left(t\right)\\ {\dot{\omega }}_{z}\left(t\right) \end{array}\right\}=\frac{1}{2}\nabla \times \dot{u}=\left(\begin{array}{c} \partial_{y}{\dot{u}}_{z}\\ -\partial_{x}{\dot{u}}_{z}\\ \frac{1}{2}\left(\partial_{x}{\dot{u}}_{y}-\partial_{y}{\dot{u}}_{x}\right) \end{array}\right),$$

For details see, for example, the classical monograph by Aki and Richards [17].

Equations (1) and (2) reveal one of the core properties of direct rotational ground motion observations: rotational ground motions can be obtained from spatial derivatives of translational ground motion (right-hand side of Equations (1) and (2)), which can be estimated as finite differences from translational sensor networks [2], equivalent to pure rotational ground motion observations with a single sensor at a single point in space (left-hand side of Equations (1) and (2)). This feature allows us to derive properties of the seismic wavefield from single-point six-degrees-of-freedom observations, for which classical three-degrees-of-freedom seismology needs networks of multiple translational sensors, so-called arrays. In many cases, such arrays are extremely expensive or even impossible to maintain. Thus, rotational ground motion sensors provide a great opportunity to enhance the scientific output of seismic observations in various geophysical environments, from active volcanoes and the bottom of the ocean to glaciers or even extraterrestrial bodies (see, e.g., refs. [7,8,18,62] in the Special Issue by Bernauer et al. [10]).

In this Special Issue, Sollberger et al. [18], provide a review of the most recent advances in the field of rotational seismology. Simonelli et al. [19] explored the possibilities of locating hypocenters of local seismic events with a combination of classical three-component translational observations and direct vertical rotation rate observations from an active ring-laser gyroscope. Teisseyre et al. [20] studied seismic rotational motions generated by multiple mining blasts with three different measuring systems. Pytel et al. [21] and Fulawka et al. [16] also examined induced seismic rotational motions in the near field of mining activities in the Lower Silesian Copper Basin region. Rossi et al. [7] proposed combining accelerometers, global navigation satellite system (GNSS) receivers, and rotational sensors with a six-component Kalman filter for structural and earthquake monitoring. They proved their concept with an experimental case study, relying on the use of an industrial six-axis robot arm on which the instruments were mounted.

The fourth group of papers of the present Special Issue are devoted to applications of rotation rate sensors in **seismic engineering**. Examining Figure 1, it can be concluded that the wave passage on the ground surface results in torsional excitations *ω_z_(t)* about the z-axis and two rocking events about the horizontal axes, causing the structure to “sway” (Figure 2).

The total structural displacements of the high structure under the rocking excitations (Figure 2) consist of “stiff” rotational contribution *q*_s_(*t*) and a respective flexible, structural response *q_f_*(*t*), as shown in Figure 3a.

However, for massive structures on compliant soils, one should not forget that the rotational response may be a direct result of soil-structure interaction (SSI). In this case, the rotational motion of a structure comes solely from horizontal excitations *u_x_*(*t*) (Figure 3b). Thus, in realistic engineering situations, the actual structural rotation about the horizontal axis is a convolution of “stiff” rotational motion *ω_y_*(*t*) (Figure 2) and rotations deriving from soil compliance (*ψ_y_*(*t*) in Figure 3b). Bonkowski et al. [22] recently computed such a rocking response of structures based on the six-component data of strong translation-rocking excitations. Substantial contributions of the rocking component in the overall seismic response were noted. The interaction of rocking excitations with horizontal translation ground motion requires careful maintenance of the sign conventions, as shown in Figure 1.

When it comes to the rotations about the vertical axis ω*_z_(t)*, the problem is slightly different. Consider a multistory structure simplified to three stories made of stiff slabs and columns, in which one story substantially differs from the other two stories in its stiffness and inertial properties, by its added two walls and eccentric mass (Figure 4).

If the structure is uniform along its height and its foundation is massless, the horizontal rotations of the story slabs *φ_z_*_1_(*t*), *φ_z_*_2_(*t*), *φ_z_*_3_(*t*) and foundation *φ_z_*_0_(*t*) are uniform and equal to the torsional excitations ω*_z_*(*t*). For axisymmetric buildings, there are no torsional vibrations if the torsional excitations do not exist. In reality, all buildings differ more or less along their height; therefore, their torsions *φ_zi_*(*t*) differ among the stories (here: *i* equals 1, 2, and 3). It should be noted that the torsions *φ_zi_*(*t*) appear even in the case of solely translational excitations *u_x_*(*t*), *u_y_*(*t*), provided the structure is asymmetric, as is the case of the simplified building structure shown in Figure 4.

The problem of measuring the torsional seismic response of buildings was the subject of two papers of this Special Issue, written by Guéguen and Astorga [22] and Guéguen et al. [23]. The paper by Murray-Bergquist et al. [6] presents a special device to measure the building story torsions to better monitor the “structural health” of the building. It should be noted that torsional seismic effects are treated seriously in seismic design because they lead to unconservative designs. For these reasons, engineers know that it is better to avoid highly unsymmetric buildings in zones of very high seismicity.

The last subject of the Special Issue is **structural health monitoring** (SHM). Rotation rate sensors can be applied for improving any measurements of structural vibrations. For example, adding rotational measurements may be beneficial in measuring the inter-story drift of buildings (see ref. [31] in [24]) or monitoring the horizontal motion of stiff structural elements, such as building stories or slabs, for which two translations and torsion are enough to describe their horizontal motion (Figure 5). The latter problem was the subject of the two aforementioned Special Issue papers [23,25]. For monitoring building vibrations, special rotation rate measuring devices are described in detail in another Special Issue paper by Murray-Bergquist et al. [6], whereas a fusion of translational/rotational sensing, appropriate for SHM purposes, is the subject of the paper by Rossi et al. [7].

As already shown in numerous analyses, and experimentally confirmed, rotation rate sensors are very useful in monitoring even small variations of the flexural stiffness of beams [24]. The first example of such an application is presented in Figure 5. In this case, by comparing rotations $\varphi_{1}$ and $\varphi_{2}$, or their velocities ${\dot{\varphi }}_{1},{\dot{\varphi }}_{2}$, one can raise early alarms during plastic hinge formation. This expectation was confirmed in preliminary research by Huras et al. [24], where local stiffness losses as small as 3.7% were detected using rotation rate sensors.

Modern rotation rate sensors, due to their ever-increasing accuracy, can be applied in direct modal analyses of bar structures, as illustrated in Figure 6, where a beam equipped with a dense array of translation/rotation sensors is presented.

Any localized damage or unusually distributed cracks (in cases of the reinforced concrete beams) may be much better sensed by the beam rotation *φ*(*x*,*t*) than by respective transversal displacements. This is particularly important for reinforced concrete beams which are cracked even in their intact state; therefore, their effective monitoring is a major research challenge. In addition, two rotation rate sensors may serve in the indirect estimation of strain variations based on the beam curvature estimation 1/ρ and spatial derivative of the beam axis:

(3) $${\dot{\varepsilon }}_{max}\left(t\right)\approx \pm \frac{h\Delta \dot{\varphi }\left(t\right)}{2\Delta x},$$

as is schematically illustrated in Figure 7.

This can be particularly important for cracked reinforced concrete beams where there is a problem in properly assessing strain and estimating the local damage (i.e., the intensity of cracks). The “averaged” strain obtained from Equation (3) between two rotation rate sensors may be a good measure of such damage to monitor. The subject of the application of modal analyses and SHM for reinforced concrete beams is studied in the paper of this Special Issue written by Bońkowski et al. [26], where rotation rate sensors are applied to extract modal shapes of the cracked reinforced concrete beams, which became unsymmetrical due to the damage inflicted on the beam, in an unsymmetric way. The literature review of paper [26] addresses the challenging problem of modal analysis and SHM of cracked reinforced concrete beams in detail.

Previous Special Issues on the topic of rotation sensing [3,4] have indicated a need to develop instruments’ fit (in terms of broadband sensitivity) for a wide range of applications, although demonstrations of the potential of six-component waveform analysis have remained mostly theoretical. The papers in this Special Issue and associated publications indicate a milestone in ground motion instrumentation as well as in structural vibration monitoring. Field-deployable rotation sensors with sufficient sensitivity now exist for geophysical applications. When it comes to the monitoring of structural vibrations, convenient, small, and accurate rotation rate sensors can easily be included in the inventory of any serious researchers acquiring vibration signals. Thus, we can expect new investigations being conducted in various fields such as earthquake physics, seismic tomography, planetary seismology, environmental geophysics, and ocean-bottom seismology, as well as earthquakes and structural engineering.

## Figures and Tables

**Figure 1:**
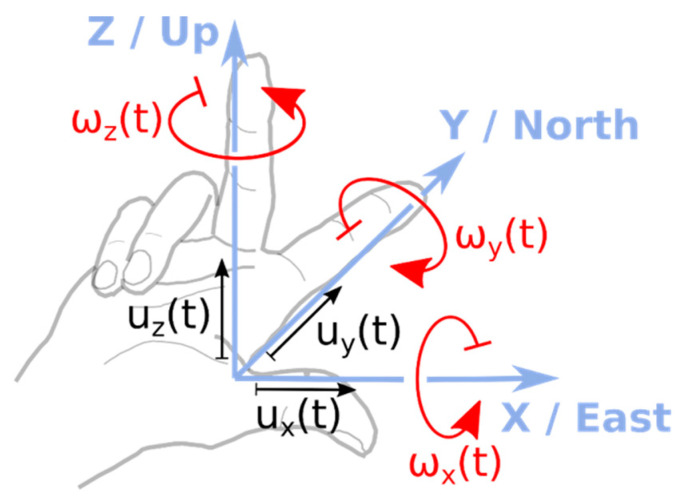
Six-component seismic ground motion system. The vertical rotation *ω_z_*(*t*) is often called *yaw*, *twist*, or *torsion*. The horizontal rotation angles *ω_x_*(*t*) and *ω_y_*(*t*) are often called *rocking*, *roll* (around the *x*-axis), and *pitch* (around the *y*-axis).

**Figure 2:**
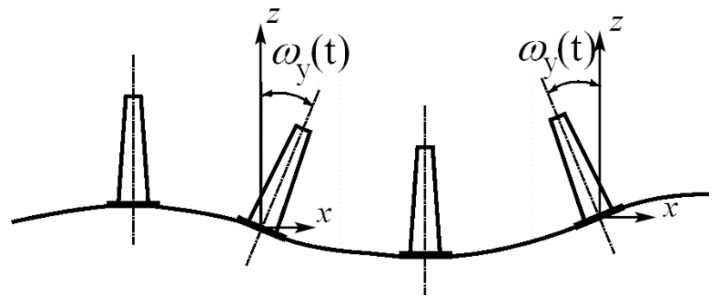
Rocking vibrations ω*_y_*(*t*) of a structure under wave passage excitations along the horizontal *x*-axis.

**Figure 3:**
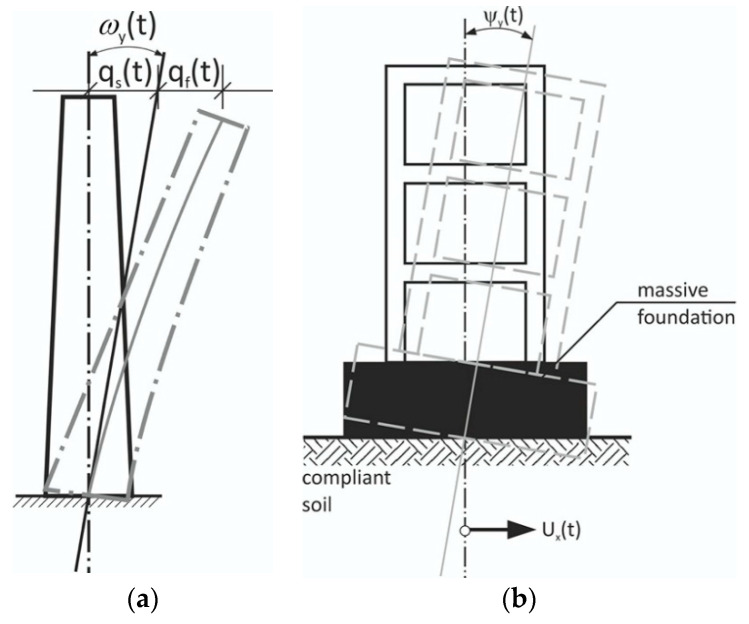
Rocking vibrations of a structure: (**a**) Slender flexible structure fixed to the non-compliant ground, under rocking excitations ω_y_(t). Its tip response *q*(*t*) consists of a contribution from the “stiff” rocking excitations and its flexible response: *q*(*t*) = *q_s_*(*t*) + *q_f_*(*t*); (**b**) A stiff structure with a massive foundation located on a compliant soil. In this case, rocking vibrations *ψ_y_*(*t*) occur even under solely horizontal excitations *u_x_*(*t*).

**Figure 4:**
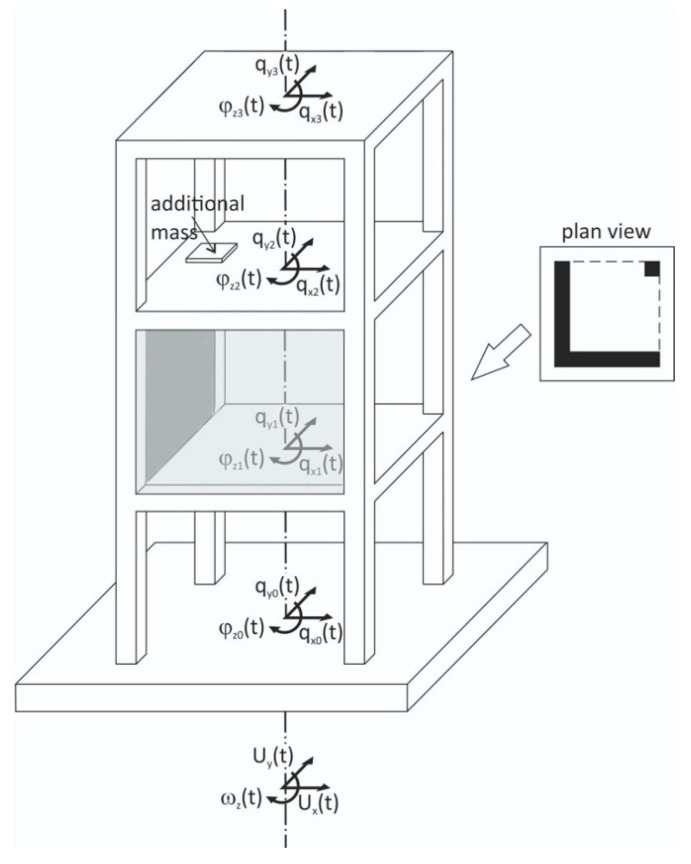
Simplified, three story, axially asymmetric building under horizontal–torsional excitations.

**Figure 5:**
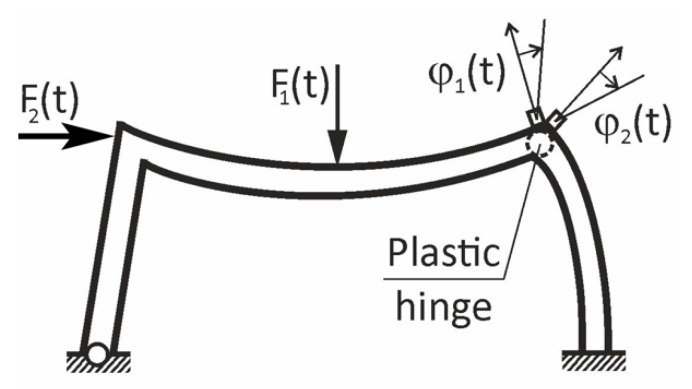
Variations of rotations of the bar ends in a frame corner, as indicators of the early stage of plastic hinge formation.

**Figure 6:**
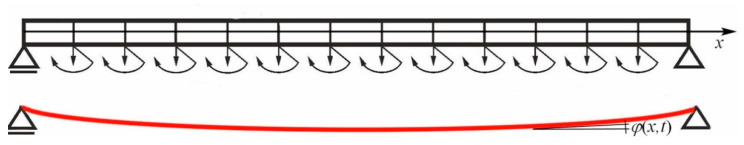
A beam equipped with translation/rotation sensors to better sense local stiffness losses in the beam axis rotations *φ*(*x*,*t*).

**Figure 7:**
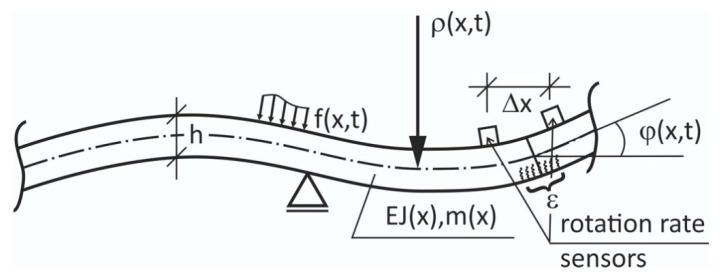
A beam with flexural, bending vibrations with curvature 1/r(*x*,*t*), with its part monitored using two rotation rate sensors at a distance of Δ*x*.
